# The Cortisol Awakening Response in Patients with Poststroke Depression Is Blunted and Negatively Correlated with Depressive Mood

**DOI:** 10.1155/2015/709230

**Published:** 2015-08-31

**Authors:** Oh Jeong Kwon, Munsoo Kim, Ho Sub Lee, Kang-keyng Sung, Sangkwan Lee

**Affiliations:** ^1^Hanbang Body-Fluid Research Center, Wonkwang University, Shinyong-dong, Iksan, Jeonbuk 570-749, Republic of Korea; ^2^Department of Psychology, College of Social Sciences, Chonnam National University, Yongbong-dong 300, Buk-gu, Gwangju 500-757, Republic of Korea; ^3^Department of Internal Medicine and Neuroscience, College of Oriental Medicine, Wonkwang University, 344-2 Shinyong-dong, Iksan, Chonbuk 570-749, Republic of Korea

## Abstract

It is important to reduce poststroke depression (PSD) to improve the stroke outcomes and quality of life in stroke patients, but the underlying mechanisms of PSD are not completely understood. As many studies implicate dysregulation of hypothalamic-pituitary-adrenal axis in the etiology of major depression and stroke, we compared the cortisol awakening response (CAR) of 28 admitted PSD patients with that of 23 age-matched caregiver controls. Saliva samples for cortisol measurement were collected immediately, 15, 30, and 45 min after awakening for two consecutive days. Depressive mood status in PSD patients was determined with Beck Depression Inventory and Hamilton Depression Rating Scale. Salivary cortisol levels of PSD patients did not rise significantly at any sampling time, showing a somewhat flat curve. Caregiver controls showed significantly higher CAR at 15 and 30 min after awakening compared to PSD patients even though the two groups did not differ at awakening or 45 min after awakening. Area-under-the-curve analysis revealed a significant negative correlation between the CAR and the degree of depression in PSD patients. Thus, our findings suggest that poststroke depression is closely related with dysfunctional HPA axis indicated by blunted CAR.

## 1. Introduction

A stroke is the rapid loss of brain function due to disturbance in blood supply to the brain. This can be due to ischemia caused by blockage or hemorrhage [1]. Stroke patients suffer from deterioration of physical ability, but in many cases emotional problems such as depression also accompany the physical symptoms. Although prevalence of poststroke depression (PSD) varies depending on the patient population, the pooled prevalence of all types of PSD is estimated to be 25–47 and 35–72% from studies carried out in patients of acute and rehabilitation stages, respectively [2]. PSD has negative impact on rehabilitation processes, but the underlying mechanisms of PSD are not completely understood [3–5]. Several epidemiological studies have shown that PSD is associated with increased disability, and poor function and cognitive outcomes in stroke survivors [6, 7]. It is observed not only in disabled stroke patients but also in those who seem to be functionally independent in their activities of daily living [8]. Thus, it is important to reduce PSD to improve the stroke outcomes and quality of life in stroke patients.

Many studies implicate dysregulation of hypothalamic-pituitary-adrenal (HPA) axis in the etiology of major depression [9]. There is accumulating evidence that dysfunction of HPA axis is not just an epiphenomenon of depression, but instead endophenotype playing a key role in its pathophysiology [10, 11]. Recent investigations have looked at associations between depression and cortisol secretion, especially, the cortisol awakening response (CAR), defined as the period of cortisol secretory activity in the first 60 minutes after awakening [12]. Some studies found significantly lower CAR in individuals with major depression [13, 14] while others found higher CAR [15, 16]. A prospective study suggests that the CAR is a better predictor of future depressive episodes when compared with other predictors [9]. Some studies suggested that an attenuated CAR may be present prior to the development of a formal diagnosis and is a biological risk factor playing a role in the pathophysiology of depression [17, 18].

Extensive studies on the relationship between cortisol secretion and stroke have found dysregulation of HPA axis function in stroke patients (e.g., [19, 20]). Also, in a recent study, we found that evening, but not morning, cortisol levels (at 8 pm) were higher in stroke patients compared to caregiver controls [21]. However, studies examining CAR in stroke patients are almost nonexistent.

Considering the involvement of HPA axis in depression and stroke, it would be interesting to see if PSD patients have altered HPA axis function. More specifically, PSD patients may show exaggerated CAR according to the general finding of hypercortisolemia in acute stroke patients, or reduced CAR according to some studies mentioned above. Thus, the present study investigated whether the CAR of PSD patients differs from a selected group of control subjects, and if so, how. Furthermore, we examined whether the magnitude of the CAR in PSD patients correlates with their depressive mood status.

## 2. Materials and Methods

### 2.1. Subjects

Participants whose stroke had happened at least 2 months before the present study were recruited from the Stroke Clinic of Wonkwang Gwangju Medical Center. Forty-nine PSD patients showing scores higher than 14 on Beck Depression Inventory II (BDI) or 7 on the Hamilton Depression Rating Scale (HDRS) were selected. Stroke was diagnosed with brain MRI. Additionally, we obtained records of neurological examinations and blood tests from all subjects. Four of these 49 patients had to be excluded because they were taking antidepressants or steroid drugs. Seventeen additional patients were excluded because of nonadherence to the sampling method or insufficient amount of saliva. Thus, the final study population consisted of 28 hospitalized stroke patients (15 males and 13 females; mean age 62.5 ± 7.9 y).

Twenty-eight age-matched patients' caregivers were initially recruited as control subjects from the same medical center. Five of these caregivers had to be excluded because of nonadherence to the sampling method (i.e., eating food or brushing teeth during sampling) or their saliva samples being reddish and contaminated with sputum. Thus, the final control population consisted of 23 caregivers (6 males and 17 females; mean age 58.2 ± 6.7 y). They lived together with their patients in our hospital. Our hospital provides space and instruments for caregivers to care for their patients closely. Thus, caregivers had schedules of daily activities similar to those of stroke patients, including sleep-awakening time, mealtime, and exercise. Caregiver controls were free of medication and did not have any neurological or psychiatric disorder at the time of testing. All participants gave informed consent. The study was approved by the Institutional Review Board of Wonkwang Gwangju Medical Center.

### 2.2. Measures

#### 2.2.1. Salivary Cortisol Collection and Assay

Since all PSD patients were hospitalized in our stroke clinic, they had very similar daily schedules for care, including the duration of rehabilitation, mealtime, and sleep-awakening schedule. They were asked to go to bed before midnight and wake up at 06:00 h. If a patient was not awake at 06:00 h on the sampling day, he or she was awakened by his or her physician or caregiver and saliva was collected according to a fixed sampling protocol [22]. Each patient provided four saliva samples: the first immediately after awakening, the second 15 min, the third 30 min, and the fourth 45 min after awakening. Patients were asked to stay in bed for the duration of saliva sampling and refrain from eating and drinking.

Caregiver controls were instructed to follow the same sampling procedures as those described above for the stroke patients. They performed the saliva sampling in the same clinic under supervision. They were also instructed to keep the same sleep-awakening schedule for several days before the sampling day and to stay in bed and refrain from eating and drinking until sampling was completed. Samples were collected by the research staff or caregivers. For both patients and controls, saliva sampling was performed on two consecutive days.

For each sample, a minimum volume of 2 mL of saliva was collected. Samples were frozen at −80°C until assay. Free cortisol in saliva samples was determined using a competitive solid-phase radioimmunoassay (Coat-A-Count, Siemens, Medical Solutions Diagnostics, Los Angeles, CA) as previously described [23]. The intra- and interassay variability were below 7 and 8%, respectively. This solid phase RIA had analytical sensitivity of 5.5 nmol/L.

#### 2.2.2. Evaluation of Depression

The Korean version of the BDI is a 21-item self-report test, one of the most widely used tools for measuring severity of depression. When presented with the BDI, subjects were asked to consider each statement as it relates to the way they have felt for the past two weeks. There is a four-point scale for each item, ranging from 0 to 3 [24]. Thus, a higher total score reflects more severe depressive mood status.

The Korean version of the HDRS is a clinician-administered 17-item multiple-choice questionnaire for rating severity of depression. It is currently one of the most commonly used scales for rating depression in medical research. Nine of the items are scored on a five-point scale, ranging from 0 to 4. The other eight items are scored on a three-point scale, from 0 to 2. A score of zero represents absence of depressive symptoms [25]. Thus, a higher total score reflects more severe depressive mood status.

BDI and HDRS were assessed on the first day saliva sampling took place.

### 2.3. Data Analysis

For each sampling point, cortisol levels were averaged for the two consecutive sampling days and analyzed using a repeated-measures ANOVA with Group as between-subject and Sampling Time as within-subject factors. Additional analysis used Scheffé test to determine the source of the detected significance. In order to obtain indices for the CAR, the global area under the curve (AUCg) for total cortisol output and the area under the response (or increase) curve (AUCi) for the responsivity of the system were computed [26]. Pearson correlation coefficients were calculated to examine whether depressive mood status (i.e., BDI and HDRS scores) was linked to the AUCg or AUCi measures of the CAR. *P* < 0.05 was considered statistically significant for all comparisons.

## 3. Results

### 3.1. PSD Patients Show a Blunted Cortisol Awakening Response

**Figure 1:**
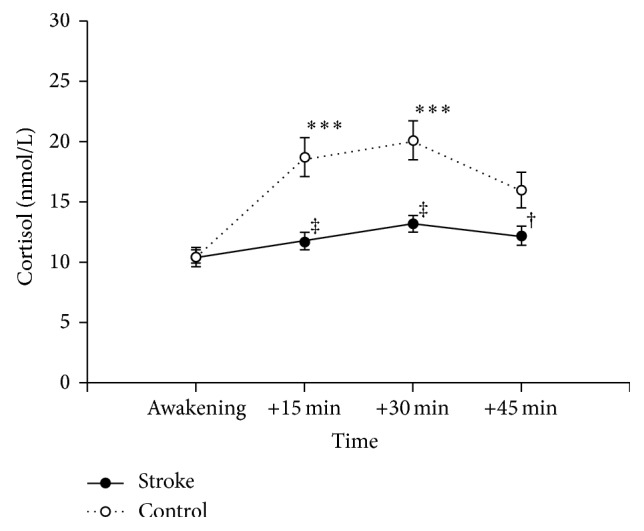
Cortisol awakening response of poststroke depression patients (stroke) and caregiver controls (control). Data points represent mean ± SEM. ^***^ *P* < 0.001, different from Awakening. ^‡^ *P* < 0.001 and ^†^ *P* < 0.03, different from caregiver controls.

Figure 1 shows salivary cortisol levels of PSD patients and caregiver controls after awakening. A repeated-measures ANOVA revealed significant main effects of Group (*F*_1,49_ = 17.095, *P* < 0.001) and Sampling Time (*F*_3,49_ = 18.775, *P* < 0.001), indicating that overall cortisol levels differed between the two groups and changed during the first 45 min after awakening. Also the Group × Sampling Time interaction was significant (*F*_3,47_ = 13.098, *P* < 0.001), indicating that the pattern of cortisol change across time differed between the groups.

At awakening, cortisol levels of PSD patients and caregiver controls did not differ. The amount of saliva cortisol of caregiver controls rose to a significantly higher level at 15 and 30 min after awakening compared to that immediately after awakening (*P* < 0.001 for both) and subsided to a marginally significant level at 45 min after awakening (*P* < 0.07). In contrast, cortisol level of PSD patients did not rise significantly at any sampling time, showing a somewhat flat curve. Increase in cortisol level only approached significance at 30 min after awakening (*P* < 0.07) in this group. Thus, while caregiver controls showed a normal CAR, the PSD patients did not.

The difference between cortisol levels of PSD patients and caregiver controls was significant at all sampling time points except for immediate postawakening (*t* = 4.148, *P* < 0.001; *t* = 4.212, *P* < 0.001; *t* = 2.333, *P* < 0.03, for 15, 30, and 45 min after awakening, resp.). Overall, this pattern of results indicates that PSD patients show a greatly blunted CAR.

Accordingly, the AUCi, reflecting the increase in cortisol level after awakening, of PSD patients was significantly smaller than that of the caregiver controls (PSD patients: 73.2 ± 21.8; caregiver controls: 311.4 ± 35.9; *t* = 5.88, *P* < 0.001). The AUCg, reflecting total cortisol output during the first 45 min after awakening, of PSD patients was also significantly smaller than that of the caregiver controls (PSD patients: 545.2 ± 22.3; caregiver controls: 782.4 ± 49.8; *t* = 4.61, *P* < 0.001).

### 3.2. The Cortisol Awakening Response of PSD Patients Correlates with Depressive Status

**Figure 2:**
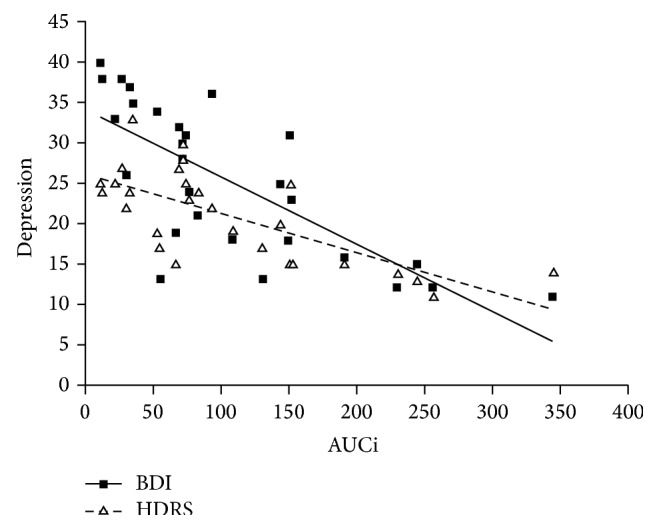
Association between the area under the response (or increase) curve (AUCi) and Beck Depression Inventory (BDI) or Hamilton Depression Rating Scale (HDRS) in poststroke depression patients (*r* = −0.747, *P* < 0.001 for BDI; *r* = −0.713, *P* < 0.001 for HDRS).

As shown in Figure 2, the AUCi for the cortisol response after awakening correlated significantly with both the BDI and HDRS scores of depressive mood status (for BDI, *r* = −0.747, *P* < 0.001; for HDRS, *r* = −0.713, *P* < 0.001), indicating that smaller AUCi values were associated with higher BDI or HDRS scores (i.e., more severe depressive status). Addition of the factors age or gender as a covariate into the analysis did not significantly alter the results.

**Figure 3:**
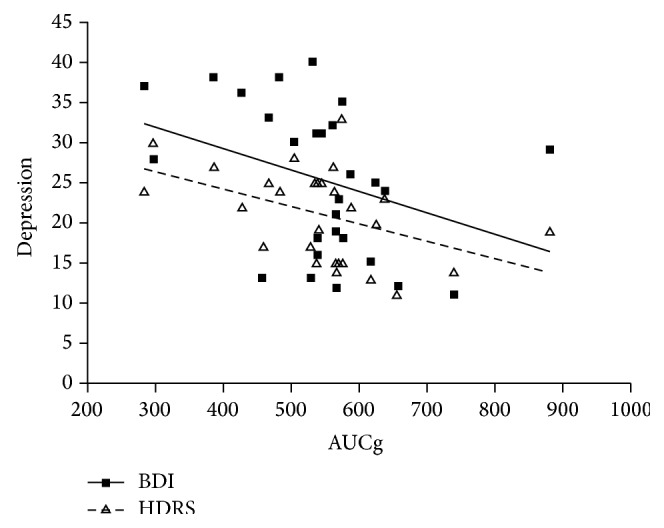
Association between the global area under the curve (AUCg) and Beck Depression Inventory (BDI) or Hamilton Depression Rating Scale (HDRS) in poststroke depression patients (*r* = −0.336, *P* < 0.1 for BDI; *r* = −0.448, *P* < 0.02 for HDRS).

The AUCg also correlated significantly with HDRS scores (*r* = −0.448, *P* < 0.02), although the correlation between AUCg and BDI scores did not reach significance (*r* = −0.336, *P* < 0.1) (Figure 3).

## 4. Discussion

The main findings of the present study are that PSD patients show a blunted CAR as compared to caregiver controls and that magnitude of the CAR is negatively correlated with severity of depression in PSD patients.

The blunted CAR in PSD patients is a novel finding, consistent with other studies showing dysfunction of HPA axis following stroke. Although cortisol secretion has been reported to be increased acutely after stroke [19, 27], our results show that, in chronic PSD patients (2 months after stroke or longer in the present study), cortisol secretion after awakening is decreased. In a previous study, we found increased evening cortisol secretion (at 20:00 h) in chronic stroke patients whose mood status was not measured [21]. Because little is known about the diurnal variation of cortisol following stroke [28], further investigations regarding this issue are needed.

The CAR is considered to be a reliable indicator of HPA axis function and has been studied extensively, not only in healthy populations, but also in relation to many disorders [22]. For example, blunted CAR has been found in chronically ill patients [29], patients with severe global amnesia [30], posttraumatic stress disorder [31], amyotrophic lateral sclerosis [32], and chronic fatigue syndrome [33]. Also, depression and emotional distress are associated with a flatter cortisol diurnal rhythm [34, 35]. Thus, the blunted CAR found in our study is indicative of dysfunctional HPA axis in PSD patients and may be related to the emotional or physical distress and PSD-associated symptoms such as fatigue, vegetative symptoms (e.g., disturbances in sleep, appetite, sexual drive, or energy metabolism), or depressive mood.

The HPA axis plays a key role in mobilizing energy resources in conditions of reduced energy supply [36]. The early morning rise in HPA axis activity originates, in part, from a negative energy balance and increases in cerebral glucose demands [37]. Stroke causes changes in the energy-dependent processes [38]. Vegetative symptoms including disturbance in energy metabolism are common in both stroke and depression patients and become worse in depressed than nondepressed stroke patients [39]. Exercise, including ambulation, is known to stimulate HPA axis activity, increasing the production of cortisol and catecholamines [40]. Thus, lack of exercise such as ambulation may induce dysfunction of the HPA axis in stroke patients, which in turn would cause the blunted CAR in PSD patients.

Although physical disability may cause reactive depressive processes in the early stages after stroke, it does not mediate the development of PSD in the long run [41]. In the present study, we recruited PSD patients 2 months after stroke or longer. The correlation between depressive mood status of our patients and AUCi and AUCg of their CAR remained significant after controlling for motor impairments (data not shown). Thus, it is not likely that the strong correlation between the blunted CAR and depressive mood status in our PSD patients is a direct consequence of physical disability.

The CAR is known to be related with depression in nonstroke patients. Participants who were currently or previously depressed [15, 42] or had higher depressive symptoms [16] exhibit higher CAR. In contrast, another study showed a blunted CAR in depressed participants [14]. The inconsistent findings may be due to differences in illness stages, as well as methodological differences in cortisol sampling [15]. A recent meta-analysis study specifically looking at the AUCi or absolute increase of cortisol secretion found a negative relationship between the CAR and severity of depression [43], which is consistent with our finding.

Experiencing a stroke can be a traumatic event. Stroke patients face enormous emotional challenge due to physical disability, profound changes in social and professional roles, and often financial insecurity. Thus, it is worth noting that many studies reported blunted CAR in posttraumatic stress disorder patients (see [43]), which is consistent with our finding.

The treatment of PSD usually involves administration of selective serotonin reuptake inhibitors or tricyclic antidepressants. However, antidepressants have not been shown to have a positive impact on neurological disturbances including cognitive deficits [44–46]. The present finding of reduced responsivity of the HPA axis correlated with depressive mood in PSD patients may provide interesting and novel information toward successful treatments of PSD. Because cortisol is related to a wide variety of functions including energy metabolism, immune function, cognition, and neuronal degeneration, restoring HPA axis function may have desirable effects on the quality of life of PSD patients.

Limitations of the present study include its cross-sectional nature and lack of comparison group without PSD.

In sum, the findings of the present study show a blunted CAR in PSD patients. The magnitude of the CAR was significantly associated with depressive mood. Future studies are needed to investigate the neurological or pathological mechanisms underlying this alteration in HPA responsivity in PSD patients.
